# An *Arabidopsis* Mitochondrial Uncoupling Protein Confers Tolerance to Drought and Salt Stress in Transgenic Tobacco Plants

**DOI:** 10.1371/journal.pone.0023776

**Published:** 2011-08-30

**Authors:** Kevin Begcy, Eduardo D. Mariano, Lucia Mattiello, Alessandra V. Nunes, Paulo Mazzafera, Ivan G. Maia, Marcelo Menossi

**Affiliations:** 1 Laboratório de Genoma Funcional, Departamento de Genética, Evolução e Bioagentes, Instituto de Biologia, Universidade Estadual de Campinas, Campinas, Brazil; 2 Departamento de Genética, Instituto de Biociências, Universidade Estadual Paulista, Botucatu, Brazil; 3 Departamento de Biologia Vegetal, Instituto de Biologia, Universidade Estadual de Campinas, Campinas, Brazil; Boston University, United States of America

## Abstract

**Background:**

Plants are challenged by a large number of environmental stresses that reduce productivity and even cause death. Both chloroplasts and mitochondria produce reactive oxygen species under normal conditions; however, stress causes an imbalance in these species that leads to deviations from normal cellular conditions and a variety of toxic effects. Mitochondria have uncoupling proteins (UCPs) that uncouple electron transport from ATP synthesis. There is evidence that UCPs play a role in alleviating stress caused by reactive oxygen species overproduction. However, direct evidence that UCPs protect plants from abiotic stress is lacking.

**Methodology/Principal Findings:**

Tolerances to salt and water deficit were analyzed in transgenic tobacco plants that overexpress a UCP (AtUCP1) from *Arabidopsis thaliana*. Seeds of AtUCP1 transgenic lines germinated faster, and adult plants showed better responses to drought and salt stress than wild-type (WT) plants. These phenotypes correlated with increased water retention and higher gas exchange parameters in transgenic plants that overexpress AtUCP1. WT plants exhibited increased respiration under stress, while transgenic plants were only slightly affected. Furthermore, the transgenic plants showed reduced accumulation of hydrogen peroxide in stressed leaves compared with WT plants.

**Conclusions/Significance:**

Higher levels of AtUCP1 improved tolerance to multiple abiotic stresses, and this protection was correlated with lower oxidative stress. Our data support previous assumptions that UCPs reduce the imbalance of reactive oxygen species. Our data also suggest that UCPs may play a role in stomatal closure, which agrees with other evidence of a direct relationship between these proteins and photosynthesis. Manipulation of the UCP protein expression in mitochondria is a new avenue for crop improvement and may lead to crops with greater tolerance for challenging environmental conditions.

## Introduction

As the population increases, there is a growing challenge to meet the global demand for food and to increase sustainability in agriculture [1]. However, crop production can be severely affected by abiotic stresses, such as salinity, drought, and temperature. These stresses lead to a series of changes in the plant that affect molecular, biochemical, morphological and physiological processes and result in deficient plant growth and development [2]. The changes caused by various stressful conditions are frequently due to a secondary stress (usually osmotic or oxidative) that perturbs the structural and functional stability of membrane proteins and disrupts cellular homeostasis [3], [4]. These changes are thus interconnected, and their effects on cellular metabolism and plant growth are similar. As a consequence, abiotic stresses often activate overlapping cell signaling pathways [3], [5], [6] and cellular responses, such as the accumulation of compatible solutes and the production of stress proteins and anti-oxidant compounds [2].

Reactive oxygen species (ROS) are produced during normal cellular metabolism. ROS can act as signaling molecules, but under stressful conditions, they damage a variety of cell components [7]. A growing body of evidence has indicated that ROS play a major role in depressing photosynthesis under stress, which ultimately leads to reduced crop productivity [8]. In addition to acting as the powerhouse of cells, mitochondria also have an important role in maintaining chloroplast function during water stress [9]. Several lines of evidence have indicated that the uncoupling protein (UCP) has a prominent role in maintaining mitochondrial function under normal and stressful conditions [10].

The UCP in eukaryotic organisms is a specialized protein that uncouples electron transport from ATP synthesis in mitochondria [11]. UCP mediates a fatty acid (FA)-dependent, purine nucleotide (PN)-inhibited proton leak across the inner mitochondrial membrane [12]. Mammalian UCPs have been studied since the 1970s [13], and until the discovery of a plant UCP (pUCP) in potato mitochondria by Vercesi et al. [10], they were thought to be a late evolutionary acquisition [11]. Since then, several genes encoding pUCPs have been identified and characterized in multiple plant species [14], [15], [16], [17]. Molecular phylogenetic analyses of UCPs from different plant and animal species suggest that these proteins diverged early, but their evolutionary history is not clear [17], [18], [19]. Both plant and animal UCPs have three conserved domains, which contain “energy transfer signatures” as well as other motifs that are specific of each group of UCPs [19]. However, in general, the members of this family display similar biochemical properties [17], [18], [19].

Although thermogenesis was initially attributed to UCPs, their widespread presence in eukaryotes suggests that this protein may have other functions, including acting as an antioxidant [17]. As mentioned earlier, ROS are one of the major components of a wide array of biotic and abiotic stresses, and mitochondria are a major intracellular source of ROS [20]. In this context, it has been demonstrated that energy-dissipating systems that increase respiratory electron transport, and consequently decrease oxidative phosphorylation efficiency, reduce the generation of mitochondrial ROS. Interestingly, UCP activity in the mitochondria is stimulated by superoxide and/or products of lipid peroxidation [21], [22], indicating that UCP-mediated mitochondrial uncoupling controls mitochondrial ROS formation through a negative-feedback mechanism. Moreover, the application of oxidative stress-promoting compounds, such as H_2_O_2_ or menadione, increased the expression of UCP coding genes in different plant species [23], [24]. Mitochondrial preparations from wheat seedlings exposed to salt (NaCl) or osmotic (mannitol) stress (moderate or severe) had increased UCP activity, suggesting that UCP plays a role in ROS detoxification [25]. Indirect evidence that UCPs counteract oxidative stress was obtained when leaves of transgenic tobacco plants that overexpress *Arabidopsis thaliana* UCP1 (AtUCP1) exhibited a lower level of damage and higher chlorophyll content than WT plants after challenge with exogenous H_2_O_2_ [26]. Arabidopsis plants lacking AtUCP1 due to a T-DNA insertion showed restricted photorespiration and lower rates of oxidation of photorespiratory glycine in mitochondria, which were associated with lower carbon assimilation by photosynthesis [27]. Together, these results suggest that pUCPs contribute to plant antioxidant defenses by reducing mitochondrial ROS production in response to stress [28].

This indirect evidence regarding the protective effect of pUCPs against oxidative stress prompted us to assess the role of these proteins in plant defense against abiotic stresses. Curiously, Arabidopsis plants containing a T-DNA insertion in the *AtUCP1* gene do not show increased sensitivity to cold or Cd^2+^, which usually cause oxidative stress, leading to the suggestion that UCPs might not be relevant for plant responses to these conditions [27]. To obtain direct evidence of the potential role of UCPs, we challenged AtUCP1-overexpressing plants [26] with salt and drought. Our data show that UCP overexpression allowed the plants to overcome the toxic effects of these stresses and that these responses were associated with a lower level of ROS in plant tissues. This broad protection associated with the dramatic effects of pUCP overexpression makes this protein a valuable tool for crop improvement.

## Results

### Overexpression of AtUCP1 improved seed germination under drought and salt stresses

**Figure 1:**
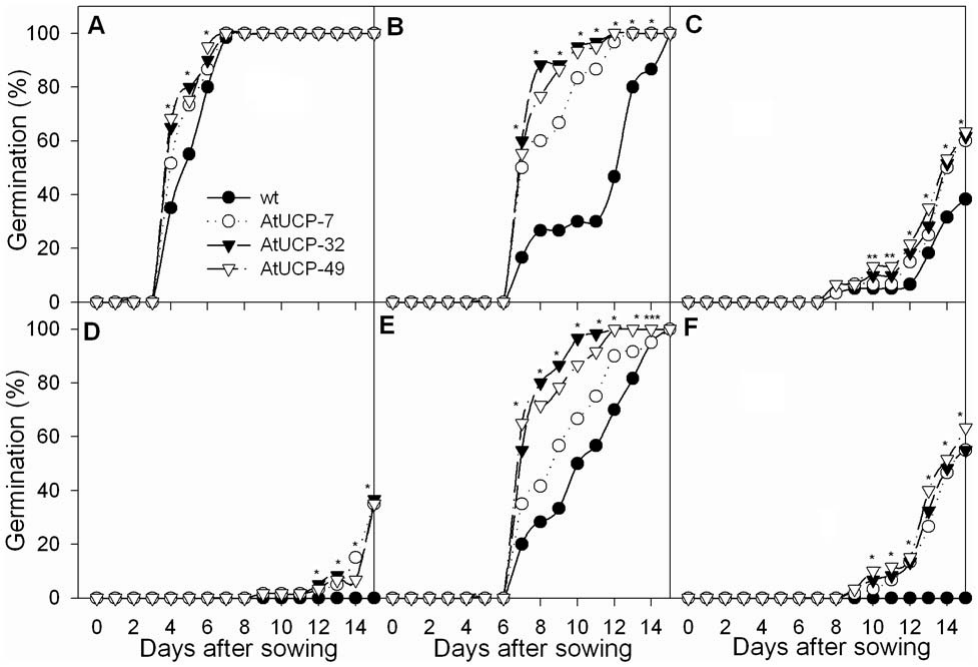
Seed germination under drought and salt stresses. Seeds from wild-type (WT) and AtUCP1 transgenic lines (7, 32 and 49) were cultivated in medium containing different concentrations of mannitol and NaCl to induce drought and salt stresses, respectively. (A) control; (B) 200 mM mannitol; (C) 300 mM mannitol; (D) 400 mM mannitol; (E) 100 mM NaCl; and (F) 175 mM NaCl. *, ** and *** indicate significant differences relative to the controls at *P*<0.0001, *P*<0.001 and *P*<0.01, respectively. The values are means of 3 independent replications (each with 30 seeds).

Seed germination depends on several environmental clues and is inhibited by drought and salt stresses. To evaluate the role of AtUCP1 in seed germination under stress, seeds from WT and transgenic tobacco plants overexpressing AtUCP1 were grown in nutrient medium containing various concentrations of either mannitol or NaCl (Figure 1). Under control conditions, WT and transgenic plants performed equally well and reached 100% germination after 8 days. Interestingly, there was a trend toward faster germination in the transgenic seeds; after 5 days, they had reached close to 80% germination, while the WT seeds were below 60% (Figure 1a). This positive effect was more evident when the seeds were exposed to mannitol stress or salt stress. At 200 mM mannitol, all of the transgenic seeds had germinated almost completely 10 days after sowing, while the control seeds did not fully germinate until day 15 (Figure 1b). As the mannitol concentration increased to 300 mM, germination of both the WT and the transgenic seeds was significantly inhibited, but the effect was more pronounced in the WT seeds (Figure 1c). At 400 mM mannitol, the germination of WT seeds was completely inhibited, while 40% of the AtUCP1 seeds were still able to germinate (Figure 1d).

The seeds from the AtUCP1 lines also showed a higher germination rate under salt stress. When 100 mM NaCl was added to the medium, the percentage of germinated transgenic seeds varied from 80% to 95%, while only 45% of WT seeds germinated after 10 days (Figure 1e). The time required to reach 50% germination was approximately 7 days for the AtUCP1 seeds and approximately 11 days for the WT seeds (Figure 1e). When challenged with 175 mM NaCl for 15 days, the difference between the AtUCP1 and WT seeds was enhanced, as 60% of the transgenic seeds germinated, while the WT seeds failed to germinate (Figure 1f). These results indicate that AtUCP1 overexpression enhanced the ability of seeds to germinate under both drought and salt stresses.

### Phenotype of transgenic plants under drought and salt stresses

**Figure 2:**
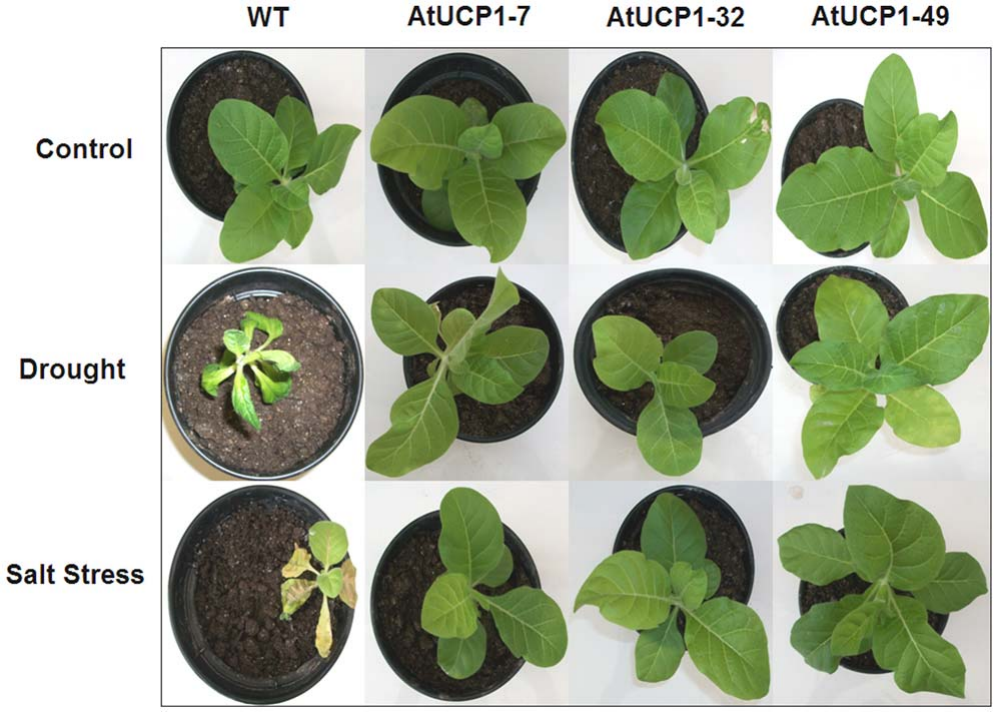
Phenotypes of wild-type (WT) and AtUCP1 transgenic tobacco plants under drought and salt stresses. First row: WT plants and three AtUCP1-overexpressing lines (7, 32 and 49) were grown under control conditions for 5 weeks. Middle row: plants watered with 200 mM mannitol for 10 days and then irrigated with water for 3 days. Bottom row: plants irrigated for 10 days with 175 mM NaCl and then irrigated with water for 3 days. A total of 5 plants from each line were used in the assay, and a representative plant is shown.

To further evaluate the response of the transgenic tobacco lines to drought and salt stresses, 5-week-old plants were irrigated with 200 mM mannitol or 175 mM NaCl for 10 days and then watered for 3 days for recovery. Control plants were irrigated with water throughout the experiment. Drought- and salt-tolerant phenotypes were clearly evident in the AtUCP1-overexpressing transgenic tobacco lines (Figure 2). The leaves of WT plants exhibited severe wilting under 200 mM mannitol, whereas those of the transgenic lines exhibited a normal phenotype. Upon severe salt stress, which killed the WT plants, the transgenic plants were able to retain a normal phenotype. These data indicate that AtUCP1 overexpression not only enhanced seed germination but also protected fully grown plants from both salt and drought stresses.

### Physiology of transgenic plants under drought and salt stresses

**Figure 3:**
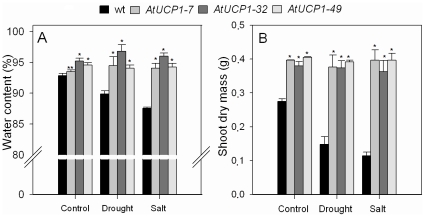
Water content (A) and shoot dry mass (B) in stressed leaves of wild-type (WT) and AtUCP1 transgenic plants (7, 32 and 49). Thirty-day-old plants were exposed for 10 days to 200 mM mannitol or 175 mM NaCl and then recovered with pure water for 3 days. In the controls, plants were irrigated with water. The bar represents the mean, and I represents the standard deviation from three independent experiments (n = 5). * and ** indicate significant differences relative to the control at *P*<0.0001 and *P*<0.001, respectively.

The water content in the leaves of the plants was evaluated after 10 days under drought and salt stress and three days of recovery with pure water (Figure 3a). Both stresses reduced the water content by 3–5% in WT plants, while plants overproducing AtUCP1 were able to maintain their water content unaffected. These results indicate that the AtUCP1-overexpressing plants were able to maintain turgidity under stress and suggest the occurrence of an osmotic adjustment and/or the activation of other defenses that prevent cellular dehydration. The effect of AtUCP1 on shoot dry mass was also evaluated. As shown in Figure 3b, WT plants had a reduction in shoot dry mass of 46% under drought and 58% under salt stress. In contrast, AtUCP1 overexpression allowed transgenic plants to maintain their shoot dry mass almost unaffected, in the range of 94–99% of plants growing under control conditions. Under stressful conditions, AtUCP1 plants accumulated more than 2-fold more shoot dry mass in drought stress and 3-fold more in salt stress than WT plants.

**Figure 4:**
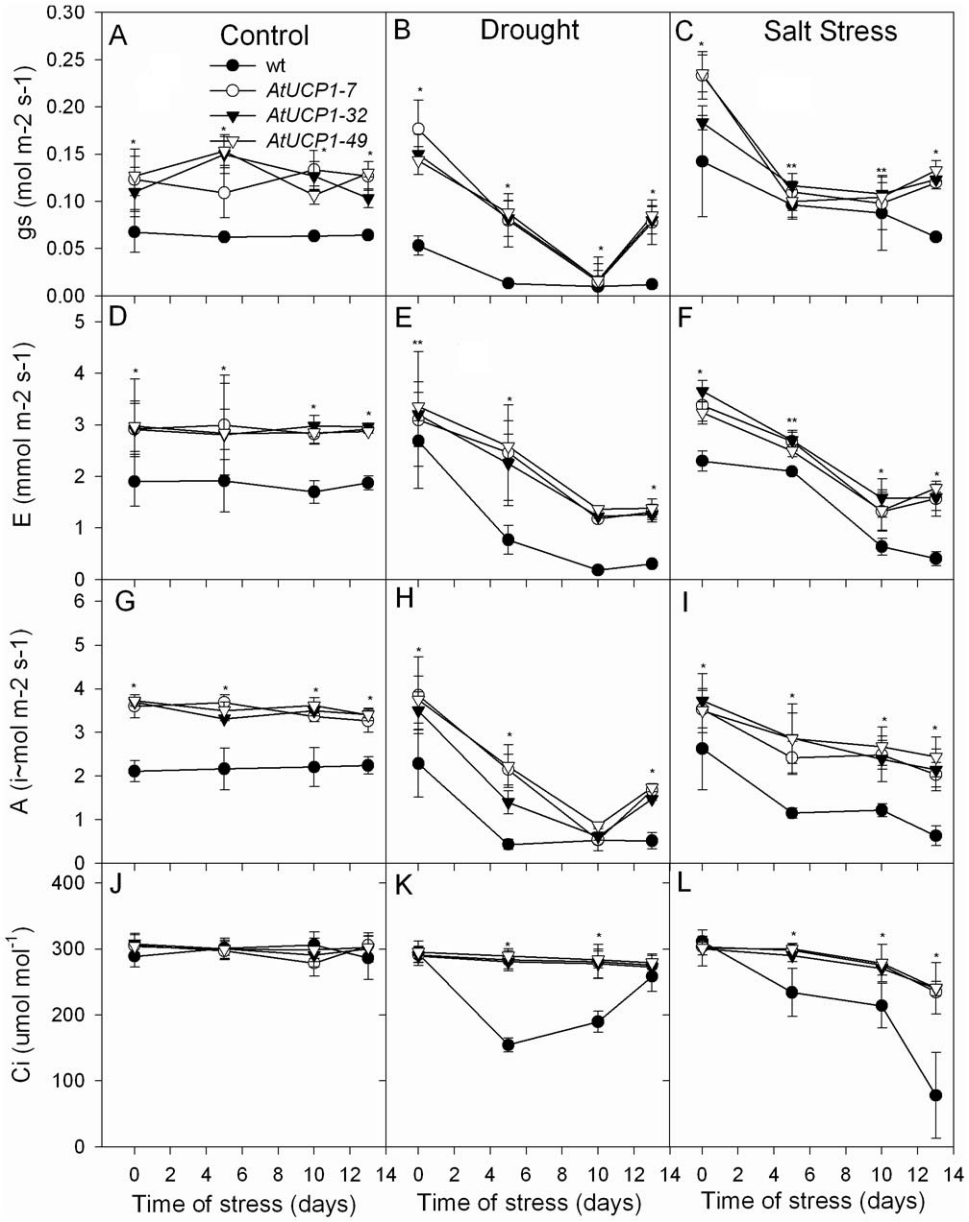
Effects of drought and salt stresses on stomatal conductance, transpiration rate, net photosynthesis and internal leaf CO_2_ concentration of wild-type (WT) and AtUCP1 transgenic plants. Thirty-day-old plants were exposed for 10 days to 200 mM mannitol or 175 mM NaCl and then recovered with pure water for 3 days. A–C: stomatal conductance (gs); D–F: transpiration rate (E); G–I net photosynthesis (A); J–L: internal leaf CO_2_ concentration (Ci). A, D, G and J: control treatment. B, E, H and K: 200 mM mannitol. C, F, I and L: 175 mM NaCl. * and ** indicate significant differences relative to the control at *P*<0.0001 and *P*<0.001, respectively.

Stomatal conductance, transpiration rate, net photosynthesis and internal leaf CO_2_ concentration were also measured in the tobacco plants after 5 and 10 days under stress and after 3 days of recovery. Under control (well-watered) conditions, AtUCP1 and WT plants exhibited no variations in any of these physiological variables throughout the experiment, as expected (Figure 4). Interestingly, the stomatal conductance (gs), transpiration rates (E) and net photosynthesis (A) were higher in the AtUCP1-overexpressing plants than in WT plants under control conditions. All of these physiological variables were negatively affected by both drought and salt stresses in both WT and transgenic plants; however, the latter generally performed better than the former for all of the parameters evaluated. The transgenic plants also recovered gs levels after they were allowed to recover from salt or drought stress (day 13 in Figure 4a–c). E also was recovered after salt stress (Figure 4f), and A was recovered after drought stress (Figure 4h) in AtUCP1 plants. Similar Ci values were found under normal growth conditions, but during most of the stress period, they were higher in AtUCP1 plants. The greatest difference between AtUCP1 and WT plants was observed for A and Ci in salt-stressed plants because the transgenic plants were able to maintain higher and more constant rates, whereas WT plants were strongly affected (Figure 4g–l).

**Figure 5:**
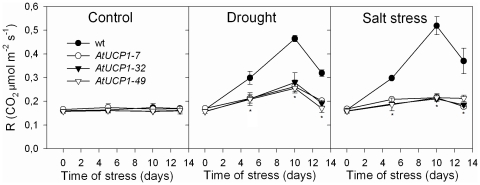
Respiration in the leaves of wild-type (WT) and AtUCP1 transgenic tobacco plants. Thirty-day-old plants were exposed for 10 days to 200 mM mannitol or 175 mM NaCl and then recovered with pure water for 3 days. Values represent the mean of three replicate measurements. * indicates significant differences relative to the control at *P*<0.0001.

Respiration measured as CO_2_ release was also evaluated during the stress and after 3 days of recovery (Figure 5). Under control conditions, no differences were observed between AtUCP1 and WT plants. The absence of changes in respiration was observed also in Arabidopsis plants overexpressing an alternative mitochondrial oxidase. However, drought and salt stress caused a marked increase in respiration in WT plants: after 10 days, respiration increased by 2.8 fold in drought stressed plants and 3.1 fold under salt stress. Even after the three days of recovery, respiration in WT plants did not returned to control levels. Respiration in AtUCP1 overexpressing plants also increased in response to these stress conditions, peaking at 1.7-fold higher on average after 10 days of drought stress and only 1.3-fold higher under salt stress. In addition to the lower increase under stress, respiration in AtUCP1 plants returned to control levels after three days.

#### Hydrogen peroxide detection in leaves of drought- and salt-stressed plants

**Figure 6:**
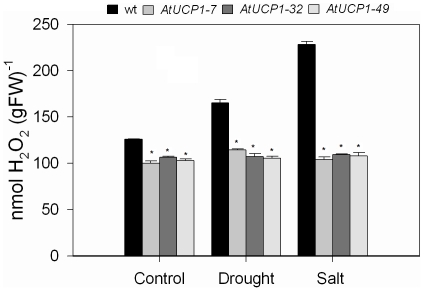
Quantification of hydrogen peroxide in leaves of wild-type (WT) and AtUCP1 transgenic tobacco plants. Thirty-day-old plants were exposed for 10 days to 200 mM mannitol or 175 mM NaCl. In the controls, plants were irrigated with water. The bar represents the mean (±S.D.) from three independent experiments (n = 5). * indicates significant differences relative to the control at *P*<0.0001. gFW: grams of fresh weight.

As hypothesized earlier, the tolerance of the AtUCP1-overexpressing lines to abiotic stresses might be related to a reduction in ROS levels. To test this possibility, the accumulation of hydrogen peroxide was evaluated in the leaves of transgenic and WT plants (30 day old) submitted to salt stress and drought stress. As shown in Figure 6 under control conditions, AtUCP1 plants showed significantly lower levels of H_2_O_2_ than WT plants (82% on average), and these levels were not affected by drought or salt stress. After 10 days of drought stress, H_2_O_2_ levels in WT plants increased by 31%, while salt stress caused a stronger effect, increasing H_2_O_2_ by 81%.

## Discussion

Enhancing plant tolerance to abiotic stresses involves multiple mechanisms and different physiological and biochemical pathways [2], [29]. Different strategies have been implemented to improve tolerance in crop plants, and transgenic plants are a powerful and promising approach. One of the major consequences of almost all environmental stresses is the appearance of secondary oxidative stress at the cellular level, and strategies aimed at increasing the antioxidant potential of plants have been shown to improve tolerance to many abiotic stresses. Because genes encoding pUCPs are induced by multiple abiotic stresses and by stress-inducing compounds [16], [24], [30], [31] and because transgenic plants that overexpress pUCPs are less sensitive to oxidative stress caused by exogenous H_2_O_2_ exposure [26], we searched for direct evidence of the protective role of AtUCP1 in plant responses to abiotic stresses.

The germination process is influenced by water availability and is critical for plant survival and early growth [32]. The final task of all metabolic, cellular and molecular mechanisms is to allow the radicle to emerge from the seed. During germination, ROS are produced by early seed imbibition; therefore, seed germination is in fact a potentially harmful process [33]. In addition, seeds rich in lipids (like tobacco) may generate ROS more actively because β-oxidation of fatty acids requires more oxygen to produce ATP [33]. Therefore, during germination, antioxidant compounds and enzymes seem to play an important role in preventing the damaging effects of ROS [33], [34]. Under drought or salt stress, seed germination may be affected, and a delay or even failure to germinate may result [35]. Because drought, salt and other stresses induce ROS production in plants [36], seed germination is expected to be more severely affected by ROS under stressful conditions. Here, we showed that transgenic tobacco plants that overexpress AtUCP1 exhibit an increased tolerance to salt and drought stresses during seed germination and early seedling development (Figure 1). Curiously, we observed that even under control conditions, the transgenic seeds germinated faster than the WT seeds, suggesting that ROS produced during germination may be attenuated by the overexpression of the AtUCP1 gene. It would be interesting to obtain seeds of transgenic crops that overexpress *UCPs* and test their longevity because the relationship between ROS and loss of seed viability has been well established [37]. This information could have economic benefits for agriculture due to an increase in seed storage time.

The protective role of AtUCP1 was also observed in mature plants challenged with drought or salt stress (Figure 2). The ability of AtUCP1-overexpressing plants to withstand these stresses was correlated with higher water levels in their leaves (Figure 3a) and higher biomass (Figure 3b). Moreover, AtUCP1-overexpressing plants exhibited higher stomatal conductance even under control conditions, and they were less affected under salt and drought stresses (Figure 4a–c). The higher stomatal conductance might be in part explained by the interaction between ROS and abscisic acid (ABA). ABA induces stomatal closure mainly by provoking an efflux of potassium and some anions from guard cells [38]. ABA-induced stomatal closure involves the production of ROS that activate Ca^2+^ influx channels in the plasma membrane [39]. Studies with *ost1* (open stomata 1) Arabidopsis mutants, which display a reduced ability to close their stomata in response to drought stress, suggest that OST1 acts in the interval between ABA perception and ROS production. Supplementation of *ost1* mutants with ABA restores stomatal closure, but the ROS levels were similar in untreated and treated mutants [40]. In our study, AtUCP1 transgenic plants showed higher stomatal conductance (greater stomatal aperture) than WT plants even under control conditions (Figure 4j–l), which could reflect the lower production of ROS in these plants. In stressed plants, the mechanism mediated by ABA and ROS might be disrupted. However, Nunes-Nesi et al. [41] found evidence that plants with mitochondrial impairments have increased stomatal closure, which in turn reduces photosynthesis. Interestingly, guard cells have an unusually high density of mitochondria [42]. The overexpression of AtUCP1 might also improve mitochondrial function, which could contribute to a greater stomatal aperture and consequently to the higher gs observed in the transgenic plants. Further studies focused on the kinases and phosphatases involved in the processes mediated by ABA and ROS [40] may provide additional information on the possible role of UCPs during stomatal closure.

When stomata are open, transpiration rates increase, which allows water to flow faster in the xylem due to a reduction in the water potential [43]. Thus, the higher water content in the transgenic tobacco plants that overexpress AtUCP1 (Figure 3) is probably a consequence of increased water flow from the roots. In the roots of plants irrigated with salt or mannitol, this phenomenon would prevent wilting. Therefore, the effect of AtUCP1 on the stomatal aperture in tobacco plants may be beneficial for the water balance of the entire plant. In fact, drought tolerance has been associated with stomatal control in plants [44].

As mentioned earlier, the tobacco plants that overexpress AtUCP1 also exhibited tolerance to salt stress (Figures 1e–f and 2). Excess NaCl imposes both ionic toxicity and osmotic stress on plants, which cause severe nutritional disorders and oxidative stress [45]. Plants with tolerance to salinity frequently have an associated capacity to extinguish ROS. Mitochondria from roots of the salt-tolerant tomato *Lycopersicon pennellii* exposed to NaCl had lower levels of H_2_O_2_ and less membrane peroxidation [46]. These plants had increased levels of ascorbate and glutathione and higher ascorbate and guaiacol peroxidase activities. Peroxisomes of this species also showed decreases in H_2_O_2_ and membrane peroxidation and increases in the activities of superoxide dismutase, ascorbate peroxidase and catalase [46]. These responses were not observed in the sensitive species *Lycopersicon esculentum*. Similar variations in the antioxidative machinery have been observed in other plant species [47]. Transgenic Arabidopsis plants that overexpress mitochondrial Mn-SOD showed significant tolerance to NaCl [48].

Drought and salinity affect photosynthesis both by altering photosynthetic metabolism and by ROS-mediated damage to the photosynthetic apparatus [49]. AtUCP1 overexpression allowed tobacco plants to exhibit higher rates of photosynthesis than wild-type plants even under control conditions, and this positive effect was also evident under both salt and drought stress (Figure 4g–i). Under salt stress, the protective effect of AtUCP1 was higher than under drought stress. However, it is interesting to note that Ci was similar in WT and AtUCP1 plants under normal conditions, indicating that increased photosynthesis in the transgenic plants is a complex metabolic process that cannot be simply explained by the increase in CO_2_ availability due to the higher gs in these plants.

The physiology that underlies the effect of AtUCP1 on photosynthesis was recently investigated [27]. These authors used an *AtUCP1* knockout mutant of Arabidopsis and found that this protein acts mainly by adjusting the bioenergetic balance of the respiratory chain during photosynthesis, which agrees with previous proposals by Vercesi et al. [17]. Plants lacking AtUCP1 not only had defects in photorespiration but also had lower photosynthetic carbon assimilation rates. Thus, our data indicate a correlation between the protective role of AtUCP1 against ROS generated by photosynthesis and tolerance to abiotic stresses. Consistent with our findings, Pastore et al. [25] proposed that pUCPs play a key role in durum wheat adaptation to drought by lowering drought-induced mitochondrial ROS formation through a feedback mechanism. Interestingly, another energy-dissipating system present in plants, the alternative oxidase (AOx) system, was also shown to lower mitochondrial ROS formation [50]. Pea protoplasts with lower cytochrome oxidase and AOx activities due to treatment with specific inhibitors had increased activities of several antioxidant enzymes, indicating that perturbations of the ability of mitochondria to maintain ROS at the optimal levels have a clear negative effect on photosynthesis [51]. These results are in agreement with the concept that energy-dissipating systems such as pUCP and AOx, which are able to tune oxidative phosphorylation, are directly involved in organelle protection against the harmful action of reactive oxygen species.

Interestingly, Rivero et al. (2007) found that delayed leaf senescence due to increased cytokinin levels caused extreme drought tolerance in transgenic tobacco plants overexpressing *IPT*, a gene encoding isopentenyltransferase, which is a key enzyme in the cytokinin biosynthesis. IPT overexpressing plants showed higher levels of photosynthesis under stress, and surprisingly, they also showed increased expression of genes involved in the control of ROS. The authors hypothesized that both factors contributed to the drought tolerance observed in the transgenic IPT plants. We believe that this effect is also the case for the AtUCP1 plants exposed to drought and salt.

The impact of abiotic stresses on plant productivity can also be due to their effects on respiration [9]. Between 30 and 70% of the CO_2_ fixed each day is released back into the atmosphere byplant respiration [9]. In water-stressed plants, the percentage of fixed carbon that is respired is predicted to be higher because, in general, drought has a greater proportional inhibition on photosynthesis than on plant respiration [8]. The effects of drought stress on plant respiration vary according to the severity of the stress and also among species [52]. The increase in respiration observed in WT plants could reflect a strategy by the plant to increase ATP levels to repair the damage caused by drought and salt stress, as demonstrated by Slot et al [53] in drought-stressed *Geum urbanum* leaves. Considering that AtUCP1 overexpression reduced the deleterious effects of abiotic stress, the need for higher respiration rates was reduced, leading to higher biomass accumulation, which might also be enhanced by the higher photosynthesis under stress. It is interesting to note that overexpression of AtUCP1 did not cause an increase in respiration under control conditions. This result probably reflects the tight regulation of the uncoupling activity of UCPs in vivo [17], [54]. Moreover, Arabidopsis plants with increased levels of a mitochondrial alternative oxidase also had no changes in total respiration rates but exhibited a reduction of ROS production [55].

The fact that AtUCP1-overexpressing plants have a higher tolerance to abiotic stresses conflicts with the absence of increased sensitivity to Cd^2+^, cold and antimycin A (a respiratory inhibitor) in plants lacking AtUCP1 activity due to a T-DNA insertion [27]. However, the activity of a combination of proteins, including AOx [56] and NADH dehydrogenase [57], which act in energy dissipation, could compensate for the lower UCP activity, as proposed by Sweetlove et al. [27]. In addition, due to the multigene nature of the UCP family in Arabidopsis [18], other UCP isoforms could also compensate for the *AtUCP1* mutation. Our data suggest that increased AtUCP1 levels provide an enhanced ability to overcome ROS overproduction under abiotic stresses.

Differences in peroxide levels, stomatal conductance, transpiration rates and net photosynthesis rates were found between wild-type and AtUCP1 transgenic plants. Therefore, a phenotypic comparison between transgenic and non-transgenic plants showed clear evidence that overexpression of AtUCP1 in transgenic tobacco plants increases tolerance to different abiotic stresses. Important differences were also noted between other parameters, such as photosynthesis, respiration, leaf water content, respiration, and these differences probably underlie the various mechanisms of tolerance. Although it was already known that overexpression of AtUCP1 in transgenic tobacco plants increases tolerance to oxidative stress caused by exogenous H_2_O_2_ [26], we obtained direct evidence for the superior performance of these transgenic plants under abiotic stresses that are known to cause ROS production. Our data highlight the protective role of pUCPs *in vivo* and provide a new approach to developing plants with enhanced tolerance to various abiotic stresses. In addition, our results suggest that transforming plants with AtUCP1 may enhance seed viability, improve the water balance in the entire plant and increase plant growth through increased photosynthesis.

## Materials and Methods

### Plant material

*Nicotiana tabacum* SR1 plants were transformed with an expression cassette comprising a double 35S promoter that controls the *AtUCP1* gene from *A. thaliana*, as described previously [26]. Three independent and homozygous lines of AtUCP1-expressing tobacco plants (AtUCP1-7, AtUCP1-32, and AtUCP1-49) were chosen for this study.

### Seed germination assays

To determine the effects of drought and salt stress on seed germination and seedling growth, seeds from transgenic and wild-type tobacco plants were used. Seeds were surface-sterilized with 70% ethanol for 1 min, incubated in 2% NaClO for 30 min and rinsed five to six times in sterile distilled water. Seeds were sown in Petri dishes (30 seeds per dish) containing solid Murashige-Skoog (MS) medium, pH 5.8, in a chamber at 23°C with a 16/8 h light/dark photoperiod (300–400 µmol photons m^−2^ s^−1^). Mannitol (0, 200, 300 and 400 mM) or NaCl (0, 100 and 175 mM) were included in the medium to induce drought or salt stress, respectively. The number of germinated seeds was counted daily; germination was defined as the emergence of the hypocotyl from day 1 to day 15.

### Gas exchange parameters measurements in tobacco plants

Seeds of the WT and AtUCP1-overexpressing lines were germinated for 16 days in Petri dishes containing MS medium at pH 5.8. Seedlings were transferred to 500-ml pots containing Plantmax HT (Eucatex, Brazil) for 5 weeks in a growth chamber at 25°C with a 16/8 h light/dark photoperiod. Plants were fertilized weekly with nutrient solutions (EPPQ, Brazil). Each plant was irrigated with 70 ml of a 175 mM NaCl solution for 10 days and then irrigated for 3 days with pure water for recovery [29]. Drought stress was performed in a similar manner but with a 200 mM mannitol solution [29].

To estimate the leaf water content, plant samples were incubated at 80°C for 24 h to evaluate their dry weight, as described previously [58]. Leaf water content was calculated as (FW−DW)/(FW^x^100), where FW is the fresh weight and DW is the dry weight. An infrared gas analyzer (IRGA - LCpro+; ADC Bioscientific, UK) was used to estimate the stomatal conductance (gs), transpiration rate (E), net photosynthetic rate (A) and internal leaf CO_2_ concentration (Ci) in completely expanded leaves from the same positions on the tobacco plants.

To measure leaf respiration, 5 weeks-old plants were grown in a chamber at 25°C with a 16/8 h light/dark period. To avoid transient metabolic activities following darkening, which is known as light enhanced dark respiration, measurements of night respiration were performed after 3 hours acclimation to darkness. Carbon dioxide production was measured with an infra-red gas analyzer (IRGA) as described by Pinelli and Loreto [59].

### Hydrogen peroxide determination

A modified ferrous ammonium sulfate/xylenol orange method was used [60]. After exposure of 30-day-old plants to different treatments (well irrigated, drought and salt stress) for 10 days, 300 mg of leaves was extracted in 1.5 ml methanol at 0°C. After being ground in a mortar, samples were centrifuged at 10,000 g for 5 min, and 500 µL of Fe(NH_4_)_2_(SO_4_)_2_ 1 mM and 200 µL of H_2_SO_4_ 250 mM were added to 100 µl of the supernatant. The reaction mixture remained in the dark for 5 minutes, and then 100 µL of 1 mM xylenol orange was added. The mixture was again brought into a dark condition for 20 minutes. The readings were taken on a spectrophotometer at 560 nm. A standard curve with known concentrations of H_2_O_2_ (0, 2.5, 5, 7.5, 10, 12.5 and 15 µM) was used as a reference.

### Statistical analysis

The mean values, standard deviation and *t*-test values were obtained with the pre-loaded software in Excel for statistical calculations (http://www.Physics.csbsju.edu/stats/t-test.html). A non-linear regression analysis was performed between RRG (dependent variable) and Al concentrations (independent variable) using the Weibull function y = 100/exp(ax)^b^ as the mathematical model.
